# Surgery for Primary Cardiac Tumors in Children: Successful Management of Large Fibromas

**DOI:** 10.3389/fcvm.2022.808394

**Published:** 2022-03-07

**Authors:** Tao Qian, Zhongshi Wu, Yifeng Yang, Li Xie, Ni Yin, Ting Lu, Can Huang, Hui Yang

**Affiliations:** ^1^Department of Cardiovascular Surgery, The Second Xiangya Hospital of Central South University, Changsha, China; ^2^National Health Commission Key Laboratory of Birth Defects Research, Prevention, and Treatment, Changsha, China; ^3^Department of Radiology, The Second Xiangya Hospital of Central South University, Changsha, China

**Keywords:** primary cardiac tumor, fibroma volume index, left ventricular dysfunction, surgery, pediatrics

## Abstract

**Background:**

Pediatric primary cardiac tumors (PCTs) are rare. Its clinical features and prognoses are not well defined. The management of asymptomatic patients with cardiac fibromas remains controversial.

**Objective:**

We aimed to examine our experience in surgical resection of pediatric PCT, with specific focuses on the management of large fibromas.

**Methods:**

This study included all the children who underwent surgical resection of PCT in our institution between December 2008 and June 2021. The last follow-up was performed between June 1st and August 26th, 2021. Kaplan–Meier method was used to estimate the postoperative survival, freedom from reoperation, event-free survival, and also related risk factors. The tumor volume and volume index (volume divided by body surface area) were measured for cardiac fibromas.

**Results:**

Of the 39 patients with median operative age of 9.5 [interquartile range (IQR): 1.2–16.5] years, 35 (89.7%) had benign tumors (fibromas for 15, myxomas for 13, and others for 7). The length and volume of fibromas were independent of age and symptoms (*P*s > 0.05). The fibroma volume index was negatively correlated with age (*P* = 0.039), with a mean value of 105 ± 70 ml/m^2^. Of the 15 patients with fibromas, 5 were asymptomatic, 4 received partial resection, 4 required transmural resection, and 4 presented postoperative left ventricular (LV) dysfunction (ejection fraction <50%). During the median follow-up period of 3.1 years and maximum of 12.5 years, adverse events included 2 early and 1 late death, 4 reoperations, 4 tumor recurrences, and 1 LV dysfunction lasting over one year. The 8-year survival, freedom from reoperation, and event-free survival rates were 90.4, 81.8, and 64.2%, respectively. Malignant tumor (*P* < 0.001) was associated with more adverse events. Transmural resection (*P* = 0.022) and larger tumor volume index than LV end-diastolic volume (*P* = 0.046) were risk factors for LV dysfunction following fibromas resection.

**Conclusion:**

Pediatric surgery for PCT can be performed with low mortalities and few adverse events. The size of cardiac fibroma in children relatively decreases with the increase of age. Larger tumor volume index than LV end-diastolic volume index and transmural tumor resection predicts postoperative LV dysfunction.

## Background

Primary cardiac tumor (PCT) is a rare disease with an incidence of 0.002–0.3% in autopsy series, and approximately 90% of which has benign nature (1). Rhabdomyomas and fibromas are the two most common benign cardiac tumors in childhood (2, 3). Both of them vary in size from a few millimeters to several centimeters. Specifically, rhabdomyomas are usually multiple and associated with tuberous sclerosis complex (TSC) (4). Cardiac fibromas are almost always solitary and tend to be stable in size (5). Huge cardiac fibromas are occasionally reported in cases (6, 7). Despite being rare in children, cardiac myxoma accounts for about 20% of surgical managed PCT in reported pediatric cohorts (8–10).

Clinical presentations of PCT are variable. The symptoms are associated with intracardiac obstruction, conduction system disturbance, valvular dysfunction, embolization, or systemic symptoms (2, 3). Rhabdomyomas have a peculiar characteristic of spontaneous regression, making the primary strategy of watchful waiting for asymptomatic patients. Patients with cardiac fibromas have a greater tendency to develop life-threatening arrhythmias, e.g., ventricular tachycardia (VT), in comparison to patients with other histotypes (11, 12). The presence of tumor-related symptoms has been well-recognized as the indication of surgical resection of intramyocardial tumors (10, 13). Patients with intracavitary tumors (mostly myxoma) generally require surgical treatment due to the high risk of embolic and cardiac complications.

Surgical tumor resection is safe and effective in eliminating these threats (8, 10, 14). Given the rarity of PCT, however, clinical data regarding its prognosis remain limited. The management of asymptomatic patients with large fibromas is poorly defined. This study aimed to examine our institutional experience in surgical resection of PCT in children, with specific focuses on the management of large cardiac fibromas.

## Methods

### Participants

The inpatient record database of the Second Xiangya Hospital was searched for records of all patients with cardiac mass. Cases that met the following criteria were included: (1) had tissue diagnosis of PCT; (2) underwent surgical resection in our institution; and (3) age at operation ≤ 18 years. Patients with inadequate or unreliable data for diagnosis were excluded. Patients with a tumor of the pericardium were excluded due to the significantly different surgical strategy. A review of medical records was approved by the Institutional Ethics Committee.

### Clinical Data

The study variables are given in Supplementary Table 1. Specifically, patients were defined as asymptomatic preoperatively in the lacking of the following presentations: (1) cardiac dysfunction, defined as left ventricular ejection fraction (LVEF) <50%, or presence of related symptoms or signs (e.g., dyspnea and edema); (2) ventricular arrhythmia; (3) embolic symptoms; (4) tumor-related significant hemodynamic impairment [mild or greater intracardiac obstruction and/or valvular insufficiency evaluated by transthoracic echocardiography (TTE)].

Tumors were classified as intramyocardial or intracavitary. Tumor length was the largest dimension of the mass measured by TTE within one month before the operation. The fibroma volume and the patient's LV end-diastolic volume (LVEDV) were retrospectively measured by a radiology professor (Hui Y) based on preoperative MRI or CT angiography (CTA) images. The fibroma volume index was then calculated by dividing the tumor volume by the body surface area. Tumor histotype was rechecked and described according to the 2015 WHO classification of tumors of the heart (15).

The early outcomes consist of postoperative hospital stay and major complications, including early death and LV dysfunction defined as TTE-measured LVEF < 50%. The adverse events during follow-up included death for any reason, reoperation for any reason, LV dysfunction lasting over 1 year, recurrence of the tumor or ventricular arrhythmia, and significant hemodynamic impairment. Event-free survival was defined as freedom from any adverse events during follow-ups. Subjects received the last clinical follow-up between June 1st and August 26th, 2021. Cardiac function status by the New York Heart Association (NYHA) classification was measured for subjects ≥10-year-old. Younger subjects were assessed for growth and development status.

### Statistics

Data were described as mean ± SD, median [interquartile range (IQR)], or frequency (%) when appropriate. Comparisons between the two groups were carried out using a two-tailed Mann–Whitney *U*-test, unpaired *t*-test, or Fisher's exact test when appropriate. Correlation coefficients (*r*) between the fibroma size and patients' age were calculated using Pearson's correlation tests. The Kaplan–Meier method was used to estimate overall survival and event-free survival rate, and freedom from reoperation. The survival curves were truncated when the number at risk is less than 10% of the original cohort. The univariate Cox regression analysis was performed to identify factors associated with time-varying adverse events (i.e., death, reoperation, and occurrence of any adverse events). Variable with a *P*-value < 0.20 became a candidate for stepwise backward multivariate Cox regression analysis. Variables were retained when *P* < 0.10. Results were reported as a coefficient, hazard ratios (HR) with 95% CIs. Similarly, the univariate and multivariate logistic regression analysis was performed to identify factors associated with postoperative LV dysfunction. Results were reported as a coefficient, odds ratios (ORs) with 95% CI. A *P*-value < 0.05 was considered significant. Data analysis was performed using IBM SPSS Statistics 23.0 (SPSS Incorporation, Chicago, IL, USA).

## Results

### Patients and Clinical Presentation

From December 2008 to June 2021, 39 children received surgical resection and tissue diagnosis of PCT in our institution. There were 35 (89.7%) benign and 4 (10.3%) malignant tumors (Supplementary Table 2). Fibroma (*n* = 15) and myxoma (*n* = 13) were the two commonest benign tumors, followed by rhabdomyoma and lipoma (*n* = 2 for each), and hamartoma of mature cardiac myocytes, capillary hemangioma, mature teratoma (*n* = 1 for each). Besides, myxofibrosarcoma (*n* = 2), rhabdomyosarcoma (*n* = 1), and papillary angioendothelioma (DabsKa tumor) (*n* = 1) were identified as malignant tumors (16).

Detailed characteristics of patients with intramyocardial tumors (*n* = 22) and intracavitary tumors (*n* = 17) are, respectively, shown in Supplementary Tables 3, 4. The median age at operation was 9.5 years (IQR: 1.2 to 16.2), including 9 (23.1%) infants <1 year of age. Patients with fibromas were significantly younger than patients with myxomas (6.5 vs. 16.2 years, *P* = 0.003; Table 1).

**Table 1:** Characteristics for 39 patients with primary cardiac tumors.

**Characteristics**	**Benign**			**Malignant** **(*n* = 4)**
	**Fibroma** **(*n* = 15)**	**Myxoma** **(*n* = 13)**	**Others** **(*n* = 7)**	
Gender (male, %)	8 (53.3%)	7 (53.8%)	5 (71.4%)	3 (75.0%)
Age (years)	6.5 (1.2, 9.5)	16.2 (10.1, 17.4)	2.0 (0.1, 17.2)	12.9 (2.5, 17.3)
Weight (kg)	25.0 (11.5, 28.0)	47.0 (33.3, 64.8)	13.0 (5.5, 54.0)	53.0 (15.1, 62.3)
**Clinical presentation (** ***n*** **, %)**				
Cardiac symptoms	3 (20.0%)	3 (23.1%)	0	1 (25.0%)
Ventricular arrhythmia	5 (33.3%)	0	0	0
Embolic symptoms	0	4 (30.8%)	0	1 (25.0%)
Hemodynamic impairment				
Valvular insufficiency	2 (13.3%)	5 (38.5%)	1 (14.3%)	0
Intracardiac obstruction	3 (13.3%)	2 (15.4%)	1 (14.3%)	3 (75.0%)
Asymptomatic	5 (33.3%)	3 (23.1%)	4 (57.1%)	0
**Preoperative workup (** ***n*** **, %)**				
Prenatal diagnosis	2 (13.3%)	0	1 (14.3%)	1 (25.0%)
Echocardiography	15 (100%)	13 (100%)	7 (100%)	4 (100%)
Computed tomography	13 (86.7%)	4 (30.8%)	4 (57.1%)	2 (50.0%)
Magnetic resonance imaging	10 (66.7%)	2 (15.4%)	3 (43%)	0
3D-print	2 (13.3%)	0	0	0
**Tumor location (** ***n*** **, %)**				
Atria	1 (6.7%)	9 (69.2%)	2 (28.6%)	3 (75.0%)
Ventricle	14 (93.3%)	3 (23.1%)	4 (57.1%)	1 (25.0%)
Multiple	0	1 (7.7%)	1 (14.3%)	0
Tumor length (mm)	52 (38, 56)	58 (38, 81)	25 (10, 51)	59 (41, 77)
**Operative characteristics**				
On-pump time (min)	116 ± 53	67 ± 32	85 ± 29	61 ± 38
Cross-clamping time (min)	88 ± 49	31 ± 28	46 ± 28	23 ± 12
Partial resection (*n*, %)	4 (26.7%)	0	1 (14.3%)	0
Transmural resection (*n*, %)	4 (26.7%)	-	0	0
Associated procedure (*n*, %)				
Valve repair /replace	1 (6.7%)	3 (23.1%)	0	0
VSD closure	0	1 (7.7%)	1 (14.3%)	0
PDA ligation	0	0	2 (28.6%)	0
SVC reconstruction	0	0	0	1 (25.0%)
**Postoperative characteristics**				
Intubation time (hours)	6 (5, 41)	9 (6, 17)	9 (6, 50)	16 (11, 23)
CICU stay (hours)	45 (21, 150)	21 (16, 27)	24 (22, 140)	42 (24, 136)
Hospital time (days)	10 (7, 16)	7 (6, 10)	7 (6, 9)	15 (10, 19)
LVEF <50% (*n*, %)	4 (26.7%)	1 (7.7%)	1 (14.3%)	0

*CICU, cardiac intensive care unit; LVEF, left ventricular ejection fraction; PDA, patent ductus arteriosus; SVC, superior vena cava; VSD, ventricular septal defect*.

As shown in Table 1, 12 (30.8%) were asymptomatic as defined. Patients with fibromas have a higher incidence of ventricular arrhythmia (non-sustained VT for 3 patients, sustained VT for 1) compared with other tumors (33.3% vs. 0). Patients with myxomas have a higher incidence of embolic symptoms (hemiplegia due to cerebral infarction for 3 patients, hemoptysis due to pulmonary embolism for 1) compared with other benign tumors (30.8% vs. 0).

### Preoperative Workup and Tumor Characteristics

**Figure 1:**
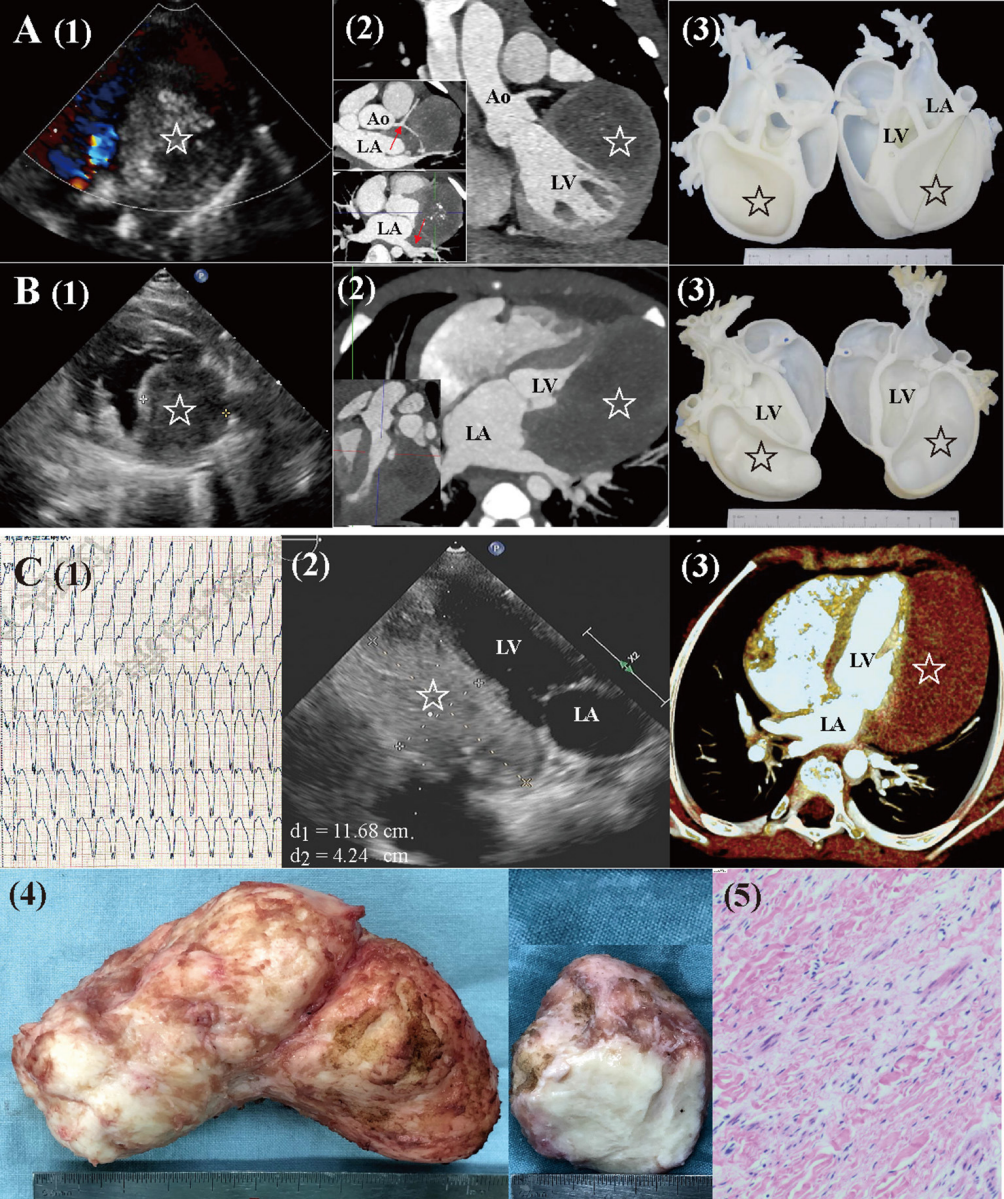
Preoperative workups and surgical resection of large cardiac fibromas. **(A)** Echocardiography, CTA, and 3D-printed models for a 4.2-year-old female. The mass (star) extended close to the left coronary artery and left pulmonary veins [red arrow in (2)], with visible calcification. **(B)** Echocardiography, CTA, and 3D-printed models for a 6-month-old male. The mass (star) extended from the base to the apex, with a distinctly larger volume than that of LV. **(C)** A 7.5-year-old male (1) presented with sustained ventricular tachycardia (176 bpm). (2, 3) Echocardiography and CTA revealed a huge mass (star. length: 12 cm, volume: 213 ml, and volume index: 227 ml/m^2^) located at LV posterolateral wall. (4, 5) Gross specimen and histologic examination of the mass, typical of fibroma. Ao, aorta; CTA, computed tomography angiography; LA, left atria; LV, left ventricle.

Magnetic resonance imaging and CTA were performed to evaluate the mass characteristics, cardiac function, and surrounding structures. Cardiac 3D-printed models were obtained for 2 patients with large fibromas to optimize surgical planning (Figures 1A,B).

**Figure 2:**
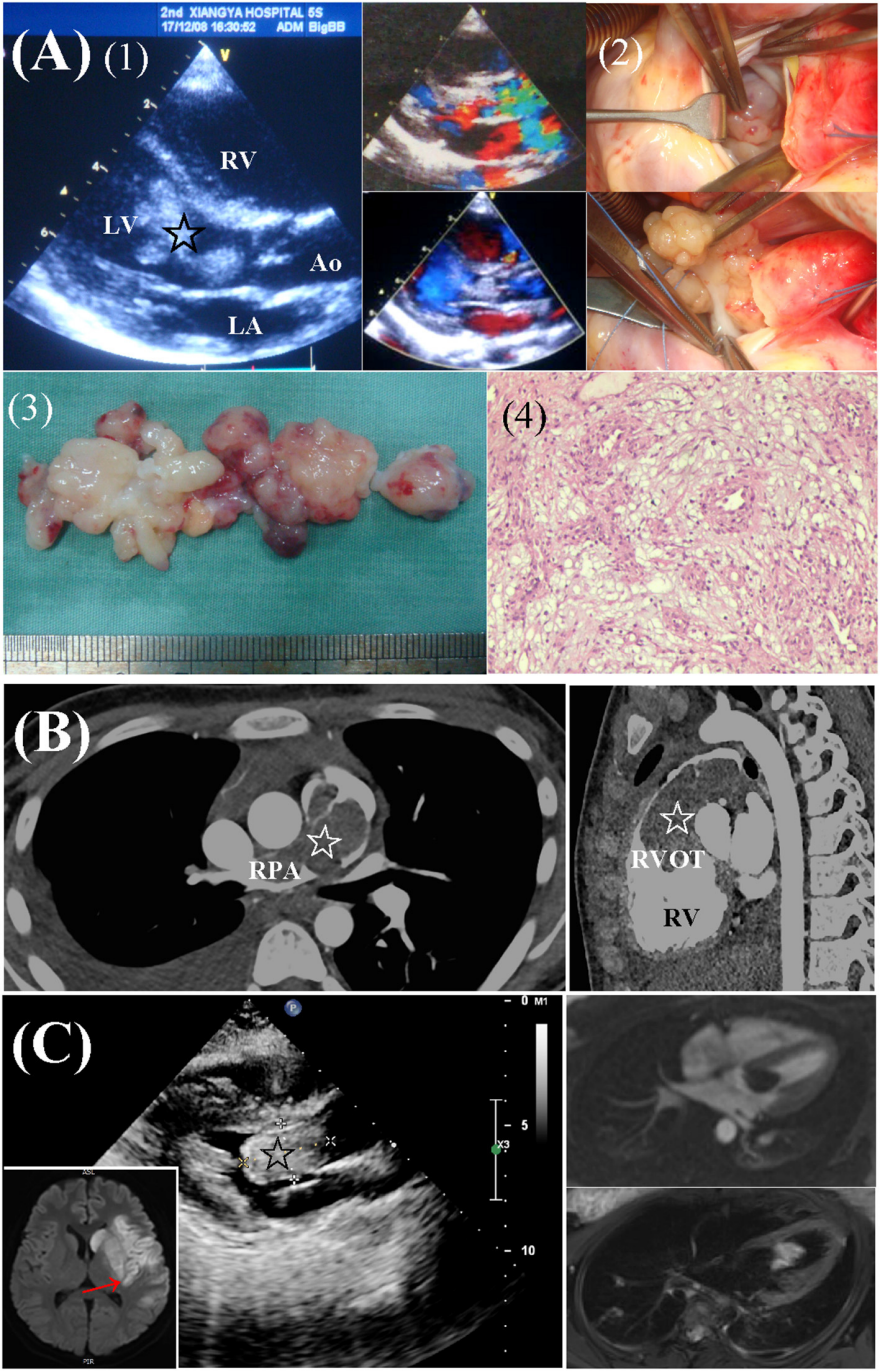
Preoperative workups and surgical resection of ventricular myxomas. **(A)** Left ventricular myxoma in a 6.2-year-old male. (1) Echocardiography revealed a lobulated, mobile mass (star) obstructing the LV outflow tract. (2) Surgical exploration and complete removal of the mass through a transverse aortic incision. (3, 4) Gross specimen and histologic examination of the mass, typical of myxoma. **(B)** A 14.5-year-old female presented with dyspnea and edema. CTA revealed a large mass (star) in RV, extending from the RVOT to branch pulmonary arteries. **(C)** A 17.2-year-old female presented with right hemiplegia and aphasia due to cerebral infarction (red arrow). Echocardiography and MRI revealed a mass (star) located in LV. Ao, aorta; CTA, computed tomography angiography; LA, left atria; LV, left ventricle; RPA, right pulmonary artery; RV, right ventricle; RVOT, right ventricular outflow tract.

Cardiac fibromas were mainly located in the wall of LV (*n* = 8), followed by interventricular septum (*n* = 4), right ventricle (*n* = 2), and left atrium (*n* = 1). Myxomas were mainly located in the cavitary of the left atrium (*n* = 5) and right atrium (*n* = 4). Ventricular myxomas were found in 3 patients (Figure 2). Multiple tumors were found in 2 patients, one with biatrial myxoma and one with rhabdomyoma and genetic diagnosed TSC.

**Figure 3:**
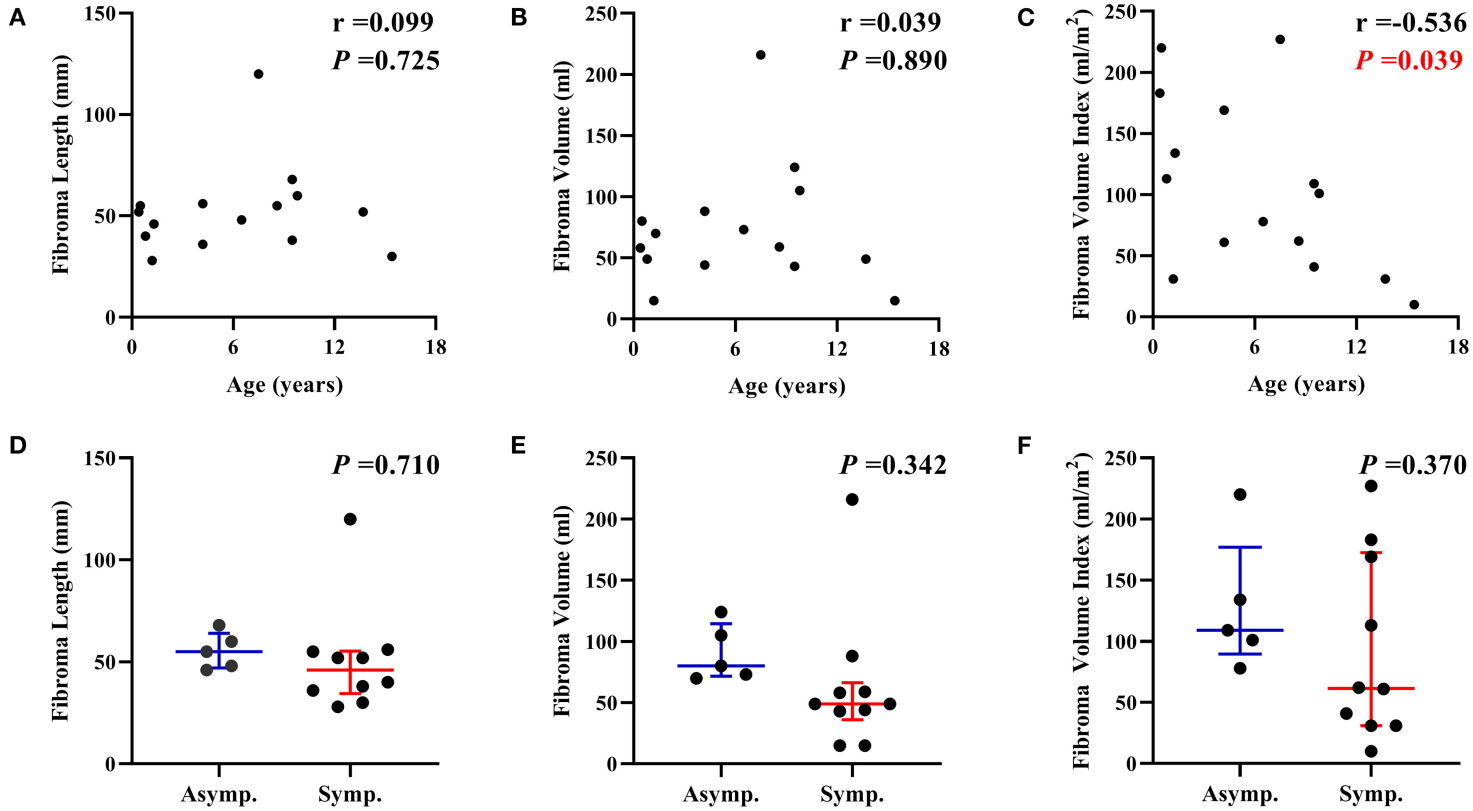
Correlation between cardiac fibroma size with age and clinical presentation. **(A)** The length and **(B)** the volume of cardiac fibromas were not correlated with age. **(C)** The fibroma volume index was negatively correlated with age. There was no significant difference between symptomatic patients (*n* = 10) and asymptomatic patients (*n* = 5) in **(D)** the length, **(E)** the volume, and **(F)** the volume index of fibromas. Asymp., asymptomatic; Symp., symptomatic.

The median length, volume, and volume index of fibromas were 52 mm (IQR: 38 to 56), 59 ml (IQR: 44 to 88), and 101 ml/m^2^ (IQR: 41 to 169; mean: 105 ± 70 ml/m^2^). The largest fibroma was shown in Figure 1C. There was no correlation between the length and volume of fibromas with age (*P*s > 0.05, Figures 3A,B). The fibroma volume index was significantly negative corrected with age (*P* = 0.039. Figure 3C). The size of the fibromas was similar between symptomatic (*n* = 10) and asymptomatic (*n* = 5) patients (*P*s > 0.05, Figures 3D–F). Symptomatic patients had similar age with asymptomatic patients (median: 4.2 vs. 6.5 years, *P* = 0.940). In total, 10 of the 15 (66.7%) patients had a larger fibroma volume index than the LVEDV index.

### Surgical Approach and Early Outcomes

The primary indication for resection of intramyocardial tumors is to alleviate tumor-related symptoms. Complete resection is the first option unless the mass is extended close to the important cardiac structures. Resections of fibromas were carried out under cardiopulmonary bypass (CPB) (mean: 116 ± 53 min) and aortic cross-clamping (ACC) (mean: 88 ± 49 min). In total, four of 15 (26.7%) fibromas were partial resected to avoid possible damage to major coronary artery (*n* = 2), atrioventricular groove (*n* = 1), and His bundle (*n* = 1). Another 4 (26.7%) patients with fibromas received transmural resection resulting in damage to the endocardium. The tumor bed was obliterated using Prolene (Ethicon Incorporation, Somerville, New Jersey, USA) suture and reinforced with felt pieces at the outer side (Supplementary Figure 1). One patient with right ventricular fibroma required concomitant De Vega's tricuspid annuloplasty.

In general, the diagnosis of an intracavitary cardiac tumor is the indication for its complete resection, especially in patients with symptoms, hemodynamic impairment, or patients with the risk of embolism. In total, six of the 17 (35.3%) patients with right atrial tumor received an operation on beating heart. The mean duration of CPB (*n* = 13) and ACC (*n* = 9) was 67 ± 32 and 31 ± 28 min for cardiac myxomas resection, both were significantly shorter than that for fibroma resection (*P*s < 0.05). Associated procedures were performed in 8 (47.1%) patients, which was more than that in patients with intramyocardial tumors (1/22 = 4.5%, *P* = 0.005).

There were 2 (5.3%) early deaths. One 5-month-old girl with LV fibroma (Supplementary Figure 2) suffered a sudden cardiac arrest on the second postoperative day. The patient required extracorporeal membrane oxygen support for 4 days after resuscitation and was died of multiple organ failure thereafter. Another newborn died because of intracranial hemorrhage 7 days after surgical removal of right atrial malignant DabsKa tumor.

Patients were discharged with uneventful courses, except 6 (15.4%) patients (4 fibromas, 1 multiple rhabdomyoma, and 1 myxoma) presented with LV dysfunction. The duration of CPB (*P* = 0.014) and underwent transmural resection (*P* = 0.008) were significantly associated with LV dysfunction in univariate analysis (Supplementary Table 5). Both the variables, however, lost significance in the multiple regression analysis (Table 2). Specifically, we examined the risk factor for LV dysfunction after resection of cardiac fibromas with a specific focus on the tumor size (Supplementary Table 6). In multivariate analysis, transmural resection (*P* = 0.022) and tumor volume index larger than LVEDV index (*P* = 0.046) are two significant risk factors (Table 2).

**Table 2:** Postoperative adverse events and significantly associated factors after resection of primary cardiac tumors (*n* = 39).

**Events**	**No. of cases (%)**	**Associated factor**	**Coef**.	**OR/HR** **(95% CI)**	***P* value**
**Postoperative LV dysfunction** ^**a**^					
All patients (*n* = 39)	6 (15.4%)	Transmural resection	2.5	11.7 (0.7, 212.1)	0.095
		CPB time	0.03	1.04 (0.99, 1.08)	0.087
Patients with fibromas (*n* = 15)	4 (26.7%)	Transmural resection	3.1	30.0 (1.4, 638.2)	0.022
		Tumor volume index >LVEDVI	1.6	5.6 (1.3, 37.9)	0.046
**Death for any reason**	3 (7.7%)	Age ≤ 1 year	4.0	56.2 (0.9, 3,399.9)	0.054
		Tumor length	0.04	1.04 (0.98, 1.09)	0.060
**Reoperation for any reason**	4 (10.3%)	None			
**Any adverse events** ^**b**^	10 (25.6%)	Malignant tumor	4.45	86.7 (7.8, 939.7)	<0.001

*CI, confidence intervals; Coef., coefficient; CPB, cardiopulmonary bypass; HR, hazard ratio; LV, left ventricular; LVEDVI, LV end-diastolic volume index; No., number of cases; OR, odds ratio*.

a *Postoperative LV dysfunction defined as LV ejection fraction < 50%*.

b *Any adverse events included death and reoperation for any reason, LV dysfunction lasting over one year, recurrence of the tumor or ventricular arrhythmia, and significant hemodynamic impairment*.

### Follow-Up Results

The last follow-up was finished in 37 (94.9%) patients. In total, one patient with myxoma and 1 patient with fibroma was lost to follow, respectively, at the 1st and 3rd postoperative years. The median duration of follow-up was 3.1 years with a range from 1 month to 12.5 years.

**Figure 4:**
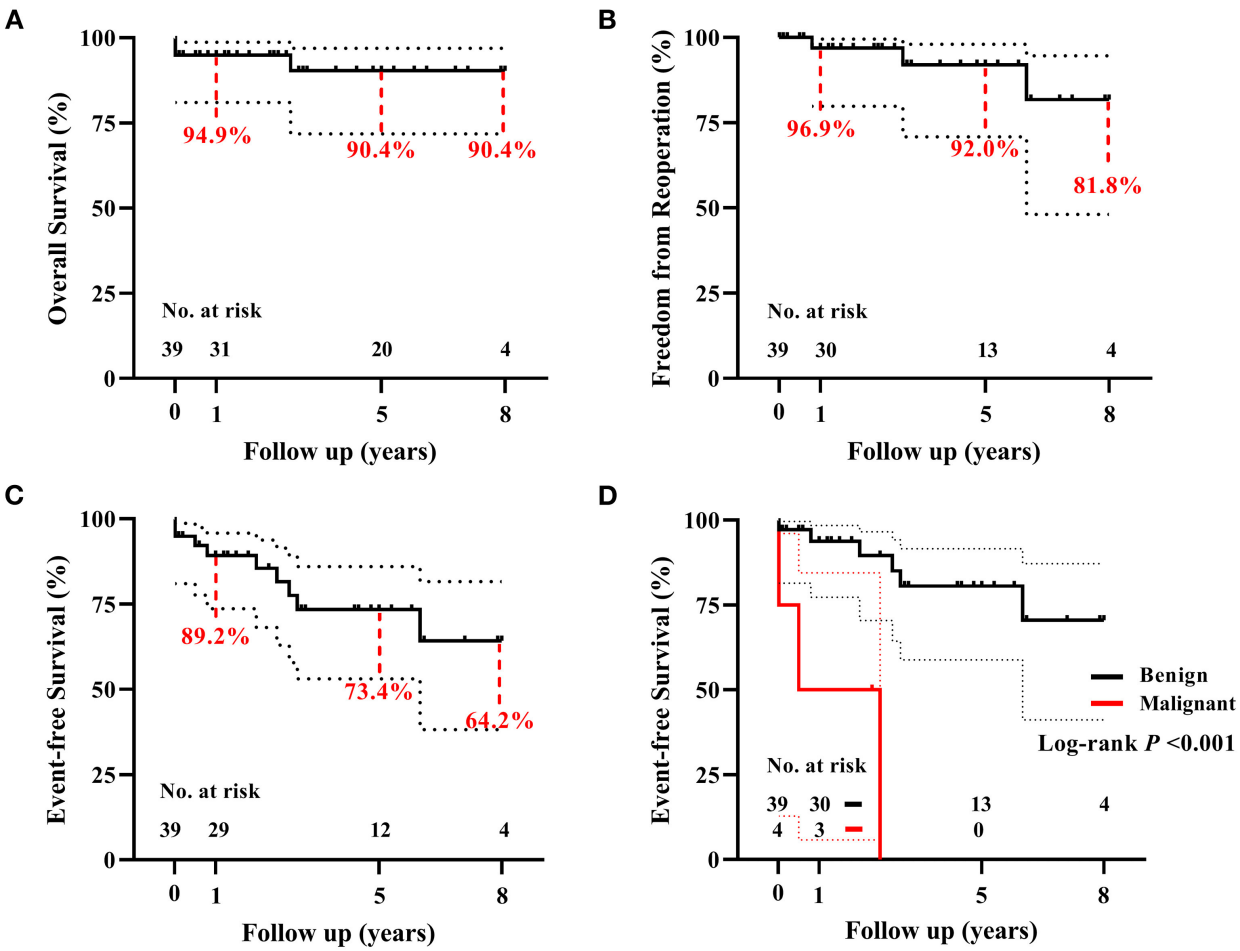
The Kaplan–Meier analyses for 39 children with primary cardiac tumors. **(A)** The overall survival, **(B)** the freedom from reoperation, and **(C)** the event-free survival following surgical resection of primary cardiac tumors. **(D)** Patients with benign tumors have a significantly higher event-free survival rate than patients with malignant tumors. Curves were truncated when the number at risk is less than 10% of the original cohort.

There was 1 (2.6%) late death. This patient presented with left hemiplegia preoperatively and died because of multiple embolisms involving the spleen and renal after 2.8 years. The Kaplan–Meier estimated survival rate was 94.9% at 1 year, and 90.4% both at 5 and 8 years (Figure 4A). There were no significant risk factors for death (Supplementary Table 5, Table 2).

During the follow-up period, 5 of 6 (83.3%) patients discharged with LV dysfunction recovered within 1 year, another one lasted for 2 years. In total, four (10.3%) patients had recorded tumor recurrence, i.e., 2 myxomas (reoperation respectively at 10 months and 3 years after primary resection), 1 poorly differentiated myxofibrosarcoma (in chemotherapy; Supplementary Figure 3), and 1 rhabdomyosarcoma (watchful waiting). Another 2 patients received reoperation for LV outflow tract obstruction, respectively, at 6 years after resection of interventricular septal fibroma, and 9 years after resection of lipoma located at the membranous septum. None of the 36 survivors had a recurrence of ventricular arrhythmia. The freedom rate from reoperation for any reason was 96.9, 92.0, and 81.8%, respectively, at 1, 5, and 8 years (Figure 4B). There were no significant risk factors for reoperation (Supplementary Table 5, Table 2). The event-free survival rate was 89.2, 73.4, and 64.2%, respectively, at 1, 5, and 8 years (Figure 4C). Histotype of malignant was significantly associated with more adverse events (*P* < 0.001, Figure 4D; Supplementary Table 5, Table 2).

At the last follow-up, one patient who underwent fibroma resection 1 month ago is taking medication for LV dysfunction (LVEF 41%); one patient is in chemotherapy for a recurrence of myxofibrosarcoma; one patient with a history of hemiplegia is in recovering of language function and physical ability; another patient is recovering in hospital after reoperation for LV outflow tract obstruction. The remaining patients are asymptomatic with the NYHA class I/II or with normal growth and development.

## Discussion

We presented a comprehensive review of the characteristics of PCT and its prognosis in 39 children. The overall survival and event-free survival rate following surgical tumor resection were 90.4 and 64.2% at 8 years. We highlight the impact of transmural resection and larger tumor size on postoperative course for patients with fibroma.

Resection of primary cardiac tumors has been proved as a safe surgical procedure with a low mortality rate. The longitudinal European multicenter study reported early mortality of 4.5% in 89 children (10). The National Inhospital Sample database in the United States recorded 186 (1.5%) in-hospital mortalities among 12,811 cases of surgically managed PCT (17). Few studies have reported long-term outcomes following PCT resection, especially in pediatrics. Nevertheless, available data all showed excellent long-term survival, with a 10- to 20-year survival rate of over 90% (10, 18, 19). In our study, the 2 cases of early deaths were both infants less than 6-month-old with demonstrable clear non-cardiac causes, corroborating the safety of surgical resection of PCT. The midterm survival rate was stable at 90% without a downward trend in the survival curve.

Patients with histologically malignant tumors usually have worse survival outcomes (10, 14). We find a significant difference in the incidence of adverse events but not in survival rate, since the relatively short follow-up for patients with malignant tumors. Complete resection is the preferred surgical strategy for cardiac tumors. Partial tumor resection (mainly for fibromas) does not affect the survival outcomes but brings more adverse events (10). Residual fibroma tissue trends to associate with recurrence of VT (20). However, partial resection does not show any influence on the prognoses in our cohort. Instead of that, we find that transmural resection is the risk factor for postoperative LV dysfunction for patients with fibromas. This finding may be attributable to the impaired endocardial blood supply and more myocardial damage in transmural resection. Therefore, it seems reasonable to avoid transmural resection even by leaving a thin layer of residual tumor tissue when resection large fibromas. This view needs further verification due to the very wide effect size (Table 2).

The management of asymptomatic patients with cardiac fibromas remains controversial. Surgical tumor resection is generally recommended to eliminate symptoms (12, 13), but is also performed in many asymptomatic patients (7, 21). Herein, 1/3 of the surgically managed patients were asymptomatic because of the concern about potentially life-threatening ventricular arrhythmias. However, we find that the presence or absence of symptoms is independent of the size of cardiac fibromas. Rather, the arrhythmias are associated with a higher degree of myocardial interdigitation within the fibroma tissue, which is negatively related to age (20). It suggests that the risk of fatal arrhythmia may decrease with age. Besides, the relative size of fibromas decreases with age in this study and others, indicating a stable or slow increase in tumor dimension (5, 20, 22). Significant larger fibroma size (assessed by fibroma length divided by physiological cardiac weight) was found for the deaths in a review study (23). In the experience from Boston Children's Hospital, fibroma volume index >100 ml/m^2^ was significantly related to postoperative LV dysfunction in the univariate analysis (22). We find that a larger fibroma volume index than an LVEDV index is significantly related to more postoperative LV dysfunction. That is, older children with relatively smaller fibroma sizes indicate a better prognosis for surgical resection, suggesting a primary strategy of watchful waiting for asymptomatic children with cardiac fibromas. Yet, the mechanism of ventricular arrhythmia in cardiac fibroma is not well understood. The establishment of clinical risk prediction models for arrhythmia, and a better understanding of the mechanism, will help us to develop individualized management strategies for children with cardiac fibromas.

Rhabdomyomas were generally reported as the commonest surgically resected primary cardiac tumors in childhood (9, 10, 19). Differently, only two children with rhabdomyomas underwent surgical resection accounting for 5.1% of our series. The lower proportion of rhabdomyomas is the result of the natural history of regression and larger age at operation than reported cohorts (8, 10, 19). Besides, most fetuses with rhabdomyomas have been aborted currently in China due to the dramatic increase of fetal detection and the high risk of combining TSC (24, 25).

The manifestations, imaging, and laboratory findings in children with cardiac myxomas are usually non-specific. We have a case of myxofibrosarcoma with multiple metastases soon after surgical resection mimicking myxoma both preoperatively and intraoperatively (Supplementary Figure 3). It may be difficult to differentiate myxomas from malignant tumors or thrombus preoperatively (26, 27). PET imaging is sensitive in ruling out malignant tumors, which is costly (28). Although it is histologically benign, cardiac myxomas have a reported recurrence rate of 1–6% (27). In this study, 2 of the 13 (15.4%) myxomas recurred postoperatively, which may attribute to the higher recurrence rate in younger patients (29).

## Study Limitation

One significant limitation of this study is the small sample size due to the rarity of the diseases, resulting in the limited validity of the multivariate analysis findings. The 95% CI for the estimated effect sizes is wide, indicating a large degree of uncertainty about the estimates. Besides, the length of follow-up is not sufficient for long-term outcome analyses. We have to truncate the survival curves at the 8th year.

## Conclusion

Surgical resection of pediatric PCT can be performed with low mortalities and low incidence of adverse events. The size of cardiac fibroma in children relatively decreases with the increase of age. A larger tumor volume index than LVEDVI and transmural tumor resection will increase the risk of LV dysfunction following resection of cardiac fibromas.

## Data Availability Statement

The original contributions presented in the study are included in the article/Supplementary Material, further inquiries can be directed to the corresponding author/s.

## Ethics Statement

The studies involving human participants were reviewed and approved by Institutional Ethics Committee of the Second Xiangya Hospital. Written informed consent to participate in this study was provided by the participants' legal guardian/next of kin.

## Author Contributions

ZW conceived of the presented idea. TQ took the lead in writing the manuscript. ZW, YY, LX, NY, and CH are cardiac surgeons contributed to the surgical treatment. TL is the perfusionist. HY is the radiologist contributed to the images analysis. All authors provided critical feedback and contributed to the final manuscript.

## Funding

This study was funded by the Major Scientific and Technological Projects for Collaborative Prevention and Control of Birth Defects in Hunan Province (2019SK1015).

## Conflict of Interest

The authors declare that the research was conducted in the absence of any commercial or financial relationships that could be construed as a potential conflict of interest.

## Publisher's Note

All claims expressed in this article are solely those of the authors and do not necessarily represent those of their affiliated organizations, or those of the publisher, the editors and the reviewers. Any product that may be evaluated in this article, or claim that may be made by its manufacturer, is not guaranteed or endorsed by the publisher.

## Supplementary Material

Supplementary Figure 1 Surgical incision and reconstruction of the left ventricle. The tumor bed of left ventricular fibromas **(A)** without transmural resection for a 6-month-old male, and **(B)** with transmural resection for a 7.5-year-old male. **(C,D)** Left ventricular reconstruction using running suture and reinforced with felt pieces at the outer side.

Supplementary Figure 2 The early death case of a 5-month-old infant with fibroma. **(A,B)** Preoperative workups revealed a large mass (star) located at the left ventricular free wall. **(C,D)** The gross photograph revealed a firm white mass, typical of fibroma. Ao, aorta; LVOT, left ventricular outflow tract; RA, right atria.

Supplementary Figure 3 A left atrial myxofibrosarcoma mimicking myxoma. **(A)** A 15.6-year-old female presented with dyspnea and right-side weakness due to cerebral infarction (black arrow). Echocardiography revealed an irregular mass with uneven density in LA. **(B)** Histologic examination revealed pleomorphic spindle fibroblast-like cells with a myxoid and fibrous background, compliant cardiac myxofibrosarcoma. **(C)** Six months after surgery, (1) MRI revealed normal heart and lung, and intracranial metastases (red arrow in 2); CT revealed that the tumor metastasized to the liver and adrenal glands (red arrow in 3, and 4).

Supplementary Table 1 Variables were collected for the patients with primary cardiac tumors.

Supplementary Table 2 Histotype of primary cardiac tumor (*n* = 39).

Supplementary Table 3 Detailed characteristics of patients with primary intramyocardial tumors (*n* = 22).

Supplementary Table 4 Detailed characteristics of patients with primary intracavitary tumors (*n* = 17).

Supplementary Table 5 Variables associated with postoperative adverse events for patients with primary cardiac tumors (*n* = 39).

Supplementary Table 6 The univariate regression analysis of variables associated with left ventricular dysfunction after cardiac fibroma resection (*n* = 15).
