# Supplementary figures and images for: Surgery for Primary Cardiac Tumors in Children: Successful Management of Large Fibromas
Source: Front Cardiovasc Med. 2022 Mar 7;9:808394. doi: 10.3389/fcvm.2022.808394 (PMC8934860; doi:10.3389/fcvm.2022.808394)

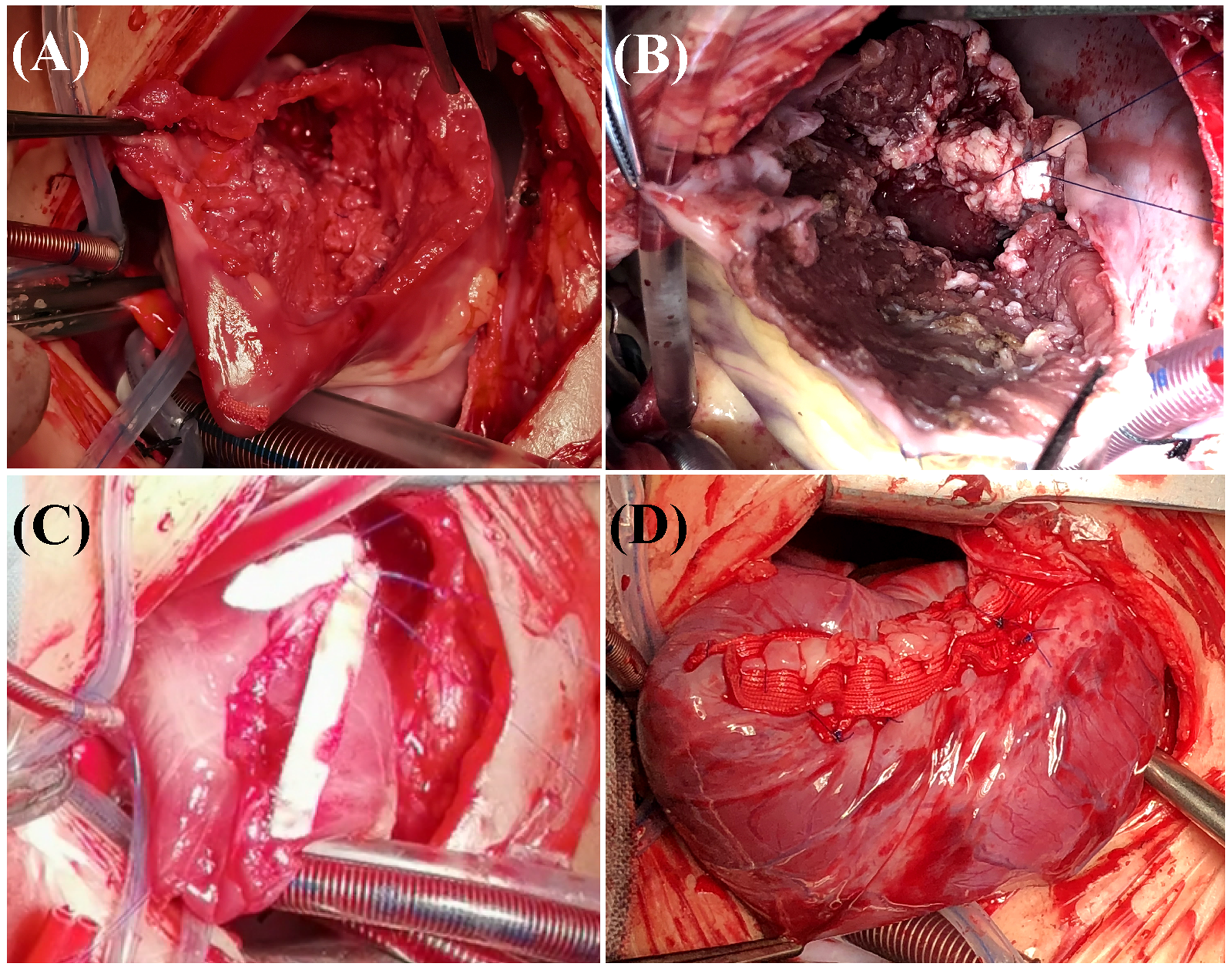

Supplement: Supplementary Figure 1 — Surgical incision and reconstruction of the left ventricle. The tumor bed of left ventricular fibromas (A) without transmural resection for a 6-month-old male, and (B) with transmural resection for a 7.5-year-old male. (C,D) Left ventricular reconstruction using running suture and reinforced with felt pieces at the outer side. [file Image_1.TIFF]

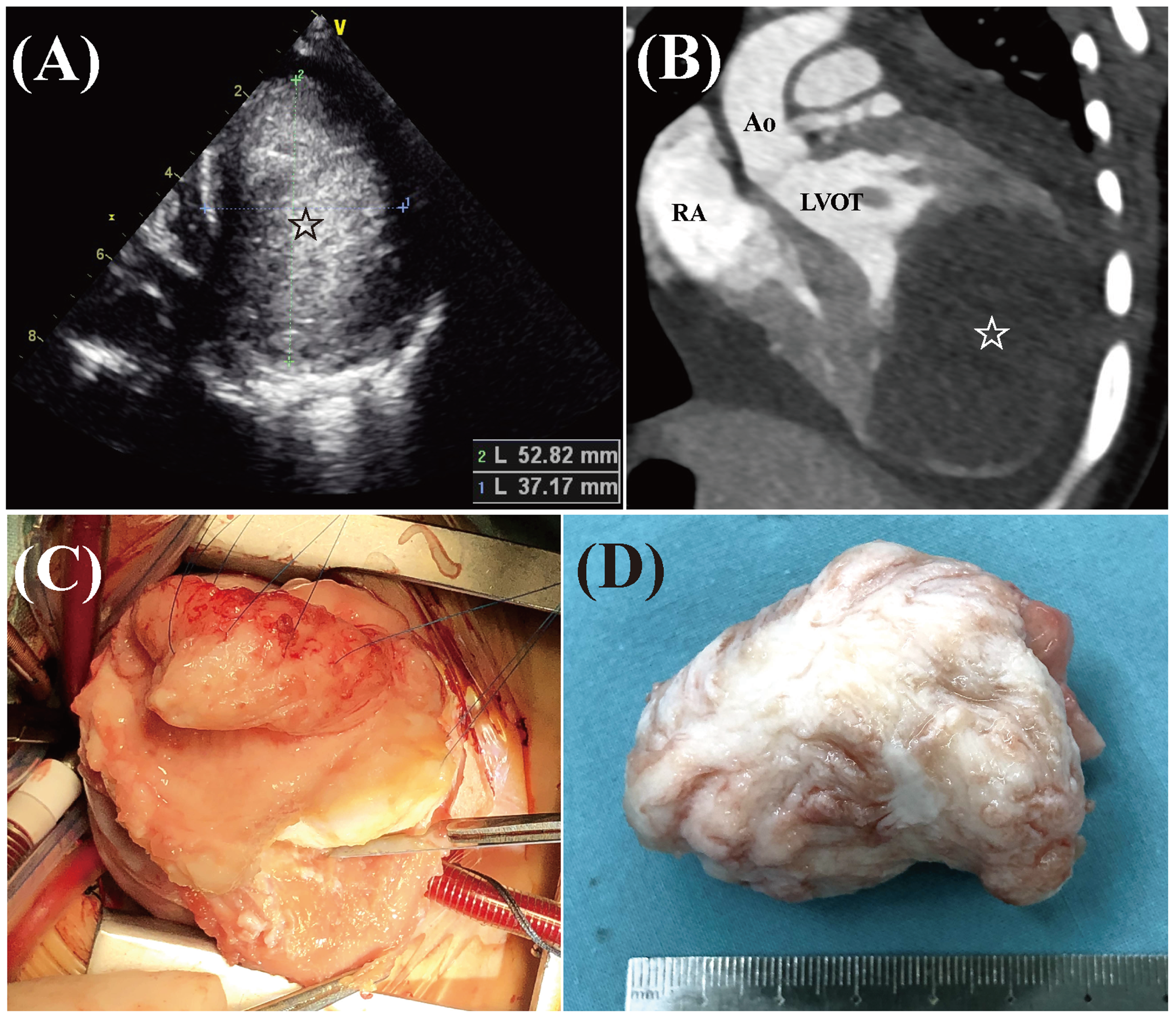

Supplement: Supplementary Figure 2 — The early death case of a 5-month-old infant with fibroma. (A,B) Preoperative workups revealed a large mass (star) located at the left ventricular free wall. (C,D) The gross photograph revealed a firm white mass, typical of fibroma. Ao, aorta; LVOT, left ventricular outflow tract; RA, right atria. [file Image_2.TIF]

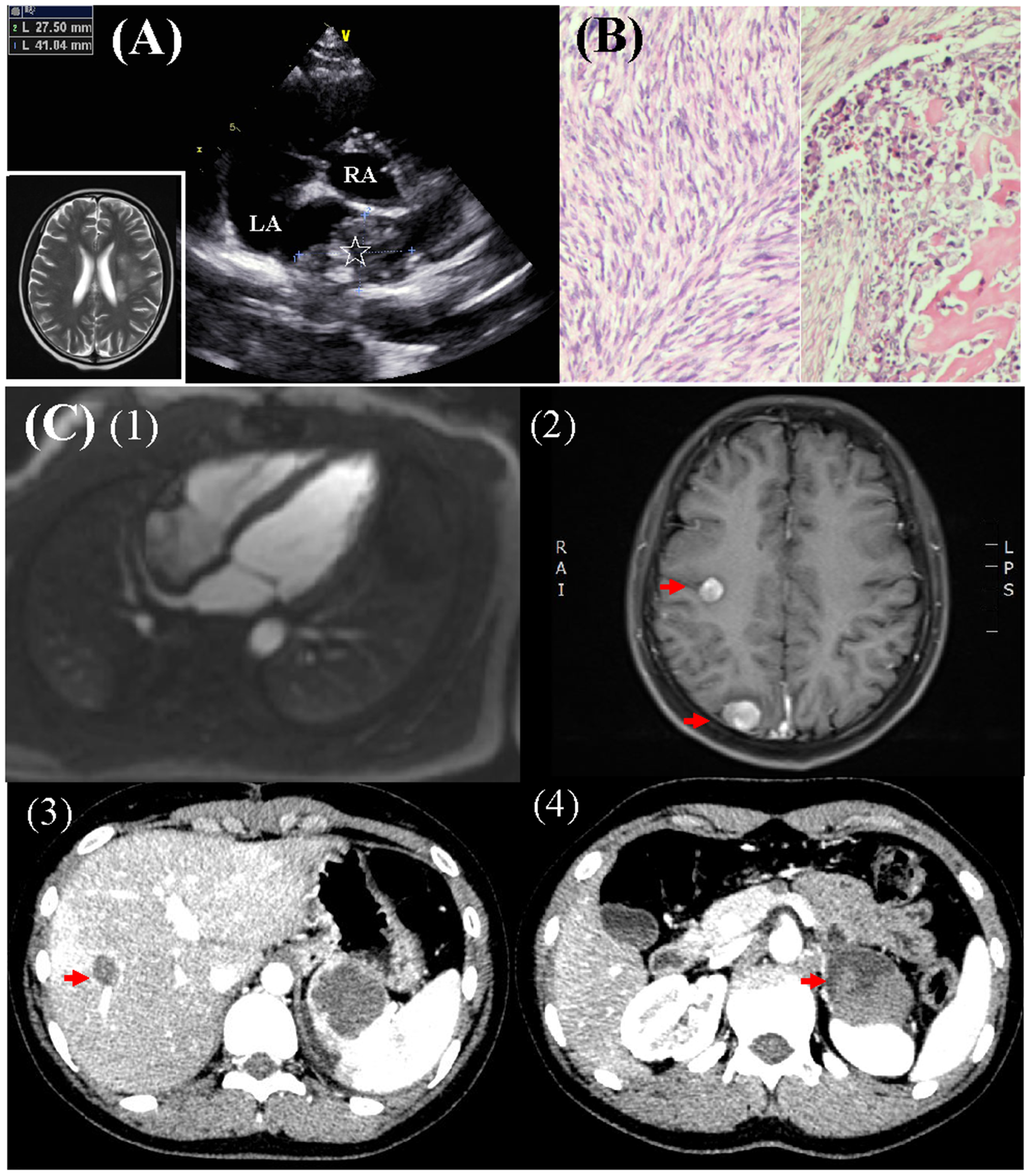

Supplement: Supplementary Figure 3 — A left atrial myxofibrosarcoma mimicking myxoma. (A) A 15.6-year-old female presented with dyspnea and right-side weakness due to cerebral infarction (black arrow). Echocardiography revealed an irregular mass with uneven density in LA. (B) Histologic examination revealed pleomorphic spindle fibroblast-like cells with a myxoid and fibrous background, compliant cardiac myxofibrosarcoma. (C) Six months after surgery, (1) MRI revealed normal heart and lung, and intracranial metastases (red arrow in 2); CT revealed that the tumor metastasized to the liver and adrenal glands (red arrow in 3, and 4). [file Image_3.TIF]
