# Supplementary material for: Surgery for Primary Cardiac Tumors in Children: Successful Management of Large Fibromas
Source: Front Cardiovasc Med. 2022 Mar 7;9:808394. doi: 10.3389/fcvm.2022.808394 (PMC8934860; doi:10.3389/fcvm.2022.808394)
Supplement: Supplementary Table 1 — Variables were collected for the patients with primary cardiac tumors. [file Data_Sheet_1.docx]

**Supplemental Table 1. Variables collected for the patients with primary cardiac tumors**

| **Demographic** | **Perioperative** | **Follow-up** |
| --- | --- | --- |
| - Gender - Date of birth - Date of diagnosis - Date of operation - Weight and height at operation | - Diagnostic workup (images, reports) - Location, size, and histotype of the tumor - Surgical strategy - On-pump and cross-clamp time - Surgical videos and photos - Major complications - Post-operative intubation, CICU stay, and hospital stay time | - Cardiac workup (images, reports) - Adverse events (date, event) - Medications - Date of last Echo follow-up - Current status (psychosomatic growth, or NYHA classification) |

CICU, cardiac intensive cure unit; NYHA, New York Heart Association.

**Supplemental Table 2. Histotype of primary cardiac tumor (n=39)**

| **Tumor histotype** | | **No. of cases (%)** |
| --- | --- | --- |
| Benign | | |
|  | Fibroma | 15 (38.5%) |
|  | Myxoma | 13 (33.3%) |
|  | Rhabdomyoma | 2 (5.1%) |
|  | Lipoma | 2 (5.1%) |
|  | Hamartoma of mature cardiac myocytes | 1 (2.6%) |
|  | Capillary hemangioma | 1 (2.6%) |
| Germ Cell Tumor | | |
|  | Teratoma, mature | 1 (2.6%) |
| Malignant | | |
|  | Myxofibrosarcoma | 2 (5.1%) |
|  | Rhabdomyosarcoma | 1 (2.6%) |
|  | Papillary angioendothelioma (DabsKa) | 1 (2.6%) |

**Supplemental Table 3. Detailed characteristics of patients with primar****y intramyocardial tumors (n=22)**

|  | **Sex** | **Age**  **at op.** | **Clinical presentation** | **Preop. workup** | **Tumor Characteristics** | | | | **Adverse events** | **Follow-up** | **Current status** |
| --- | --- | --- | --- | --- | --- | --- | --- | --- | --- | --- | --- |
|  |  |  |  |  | **Location** | **Length(mm) ^a^** | **Resection strategy** | **Histotype** |  |  |  |
| **1** | F | 5 m | NSVT (170 bpm)  PE (11mm ±); MR (mild) | TTE, MRI, CTA | LV free wall from the apex to 2/3 of distance to base | 52 | Complete  Transmural | Fibroma | Cardiac arrest (ECOM-CPR), MSOF | 6 d | Death |
| **2** | M | 6 m | Found by fetal Echo  Asymptomatic | TTE, MRI, CTA  3D-Printing | LV posterolateral wall extending from the base to apex | 55 | Complete | Fibroma | None | 6 m | Normal growth and development |
| **3** | M | 9 m | Heart failure  Dyspnea; Facial edema  SVC obstruction | TTE, MRI, CTA | Atrial septum extending from the bottom to atrioventricular valve, bulging into bi-atria | 40 | Partial (2/3) | Fibroma | None | 8.4 y | Normal growth and development |
| **4** | M | 14 m | Found by fetal Echo  NSVT (168 bpm) | TTE, CTA | Middle of IVS | 28 | Complete | Fibroma | None | 1.8 y | Normal growth and development |
| **5** | M | 16 m | Incidentally found  Asymptomatic | TTE, CTA | Anterior wall of LV, closely associated with LADCA | 46 | Partial (1/2) | Fibroma | None | 3.1 y | Lost to follow |
| **6** | F | 4.2 y | NSVT (148 bpm) | TTE, CTA, MRI  3D-Printing | LV posterolateral wall, cross atrioventricular groove | 56 | Complete  Transmural | Fibroma | LV dysfunction **^b^** (EF 41%) | 1 m | Medication |
| **7** | F | 4.2 y | Edema (lower extremities)  LVOTO (V_max_ 3.8m/s) **^c^**  RVOTO (V_max_ 2.2m/s) | TTE, MRI | Base of IVS, bulging into bi-outflow tracts | 36 | Complete  Transmural | Fibroma | None | 4.4 y | Normal growth and development |
| **8** | M | 6.5 y | LVOTO (V_max_ 2.0m/s)  Asymptomatic | TTE, CTA | Base of IVS, bulging into bi-outflow tracts | 48 | Partial (2/3) | Fibroma | Reoperation for LVOTO (V_max_ 3.8m/s) after 6 years | 7.1 y | NYHA class I |
| **9** | M | 7.5 y | SVT (176 bpm) | TTE, CTA | LV posterolateral wall extending from the base to apex | 120 | Complete  Transmural | Fibroma | LV dysfunction for 1.5 years | 2.4 y | NYHA class I |
| **10** | F | 8.6 y | Chest pain | TTE, CTA, MRI | LV free wall from the apex to 1/2 of distance to base | 55 | Complete | Fibroma | None | 5.3 y | NYHA class I |
| **11** | F | 9.5 y | Incidentally found  Asymptomatic | TTE, MRI | Middle of IVS | 68 | Complete | Fibroma | None | 1.2 y | NYHA class I |
| **12** | M | 9.5 y | Dyspnea; Chest pain Syncope | TTE, MRI, CTA | Apex of RV, extending to IVS | 38 | Complete | Fibroma | None | 6 m | NYHA class I |
| **13** | F | 9.8 y | MS (V_max_ 1.5m/s)  Asymptomatic | TTE, MRI, CTA  PET-CT | LV posterolateral wall extending from the base to apex | 60 | Partial (1/3) | Fibroma | None | 2.5 y | NYHA class I |
| **14** | M | 13.7 y | Syncope | TTE, MRI, CTA | LV posterolateral wall | 52 | Complete | Fibroma | None | 7.1 y | NYHA class I |
| **15** | F | 15.4 y | Edema (lower extremities)  NSVT (178 bpm)  TR (severe) | TTE, CTA | Anterior wall of RV, bulging into the inflow tract | 30 | Complete | Fibroma | LV dysfunction for half a year | 6.1 y | NYHA class II |
| **16** | M | 3 m | MS (V_max_ 1.8m/s)  TSC (genetic diagnosed)  Asymptomatic | TTE, MRI | Multiple (posterior wall of LA, abutting MV annulus; IVS; and the apex of RV) | 12; 9; 8 | Complete | Rhabdomyoma | LV dysfunction for 1 year | 7.9 y | Normal growth and development |
| **17** | M | 3.9 y | RVOTO (V_max_ 1.5m/s)  Asymptomatic | TTE, MRI, CTA | Anterior wall of RV, bulging into RVOT | 34 | Almost complete | Rhabdomyoma | None | 6.8 y | NYHA class I |
| **18** | F | 17.2 y | Incidentally found  Asymptomatic | TTE, CTA | LV posterolateral wall proximal to apex, outwardly growth | 51 | Complete | Teratoma, mature | None | 3 m | NYHA class I |
| **19** | M | 17.6 y | Dyspnea  PE (large, 65mm±)  TR (mild) | TTE, MRI, CTA | RA posterolateral wall, close to the orifice of SVC | 60 | Complete | Hamartoma of mature cardiac myocytes | None | 1.5 y | NYHA class I |
| **20** | M | 10.1 y | Dyspnea  MS (V_max_ 2.5m/s) | TTE | Posterior wall of LA, bulging into LA | 72 | Complete | Rhabdomyosarcoma | Tumor recurrence after 2.5 year | 3.9 y | NYHA class I  Asymptomatic |
| **21** | F | 15.6 y | 1 month after ACI (right side weakness)  PVs obstruction (V_max_ 1.8m/s) | TTE, CTA | Posterior wall of LA, occupying half of LA | 45 | Complete | Myxofibrosarcoma  (poor differentiated) | Tumor recurrence after 6 months | 1.3 y | In chemotherapy **^d^**  NYHA class II |
| **22** | F | 17.8 y | Dyspnea; SVT (125 bpm)  PE (large, 35mm ±) | TTE, CTA,  PET-CT | Surface of RV anterior wall, involving the pericardium | 40 | Complete | Myxofibrosarcoma  (well differentiated) | None | 2.3 y | NYHA class I |

ACI, acute cerebral infarction; CTA, computed tomography angiography; ECMO-CPR, extracorporeal membrane oxygenation following cardiopulmonary resuscitation; EF, ejection fraction; IVS, interventricular septum; LA, left atria; LADCA, left anterior descending coronary artery; LV, left ventricle /ventricular; LVOTO, left ventricular outflow tract obstruction; MR, mitral regurgitation; MRI, magnetic resonance imaging; MSOF, multiple systemic organ failure; MS, mitral stenosis; MV, mitral valve; NSVT, non-sustained ventricular tachycardia; NYHA, New York Heart Association; Op., operation; Preop., pre-operation; PE, pericardial effusion; PET-CT, positron emission tomography computed tomography; PVs, pulmonary veins; RA, right atria; RV, right ventricle /ventricular; RVOTO, right ventricular outflow tract obstruction; SVC, superior vena cava; SVT, sustained ventricular tachycardia; TTE, transthoracic echocardiography; TR, tricuspid regurgitation; TSC, tuberous sclerosis complex.

**^a^** Tumor length is the largest dimension of the mass measured by two-dimensional ultrasound performed within one month prior to operation.

**^b^** Postoperative LV dysfunction is defined as TTE-measured LVEF <50%.

**^c^** V_max_ is the maximum blood flow velocity measured by Doppler ultrasound.

**^d^** Chemotherapy protocol of Liposomal Doxorubicin plus Ifosfamide

**Supplemental Table 4.** **Detailed** **characteristics of patients with primary intracavitary tumors (n=17)**

|  | **Sex** | **Age at op.** | **Clinical presentation** | **Tumor Characteristics** | | | **Associated** **procedure** | **Follow-up** | **Adverse events** | **Current status** |
| --- | --- | --- | --- | --- | --- | --- | --- | --- | --- | --- |
|  |  |  |  | **Location** | **Length**  **(mm) ^a^** | **Histotype** |  |  |  |  |
| **1** | M | 20 d | Premature birth (35w)  Cardiac arrest for asphyxia | RA | 30 | Myxoma | None | 9.6 y | None | NYHA class I |
| **2** | F | 7 m | Intraoperatively found  VSD (bidirectional shunt)  Asymptomatic | RA | 5 | Myxoma | VSD closure | 2.7 y | Reoperation for tumor recurrence after 10 months | Normal growth and development |
| **3** | M | 6.2 y | LVOTO (V_max_ 3.5m/s) **^b^** | LV | 45 | Myxoma | None | 12.5 y | None | NYHA class I |
| **4** | M | 13.9 y | Chest tightness; ST (130 bpm)  MR/TR (mild) | LA | 43 | Myxoma | None | 6 m | None | NYHA class I  Trivial MR |
| **5** | F | 14.5 y | Edema (face and lower extremities); Dyspnea; ST (125 bpm)  RVOTO (V_max_ 4.5m/s); TR (severe) | RV | 150 | Myxoma | None | 5.8 y | None | NYHA class I |
| **6** | M | 16.1 y | 2 months after ACI  Left hemiplegia  Multiple arterial embolism | LA | 100 | Myxoma | None | 2.8 y | Multiple embolism (spleen, renal) | Death |
| **7** | F | 16.2 y | Chest tightness | LA | 63 | Myxoma | None | 10 m | None | NYHA class I |
| **8** | F | 16.7 y | Edema (lower extremities); Dyspnea  MR/TR (both moderate) | Biatrial | 40; 26 | Myxoma | TV valvuloplasty | 5.0 y | None | NYHA class I  TR/MR (both trivial) |
| **9** | M | 16.8 y | Incidentally found  Asymptomatic | LA | 65 | Myxoma | None | 6.1 y | Reoperation for tumor recurrence after 3 years | NYHA class I |
| **10** | F | 17.2 y | 10 days after ACI  Right hemiplegia; Aphasia  MR (severe)/ TR (moderate) | LV | 36 | Myxoma | MV replacement  TV valvuloplasty | 3.2 y | LV dysfunction for half a year **^c^** | MS (V_max_ 2.0 m/s)  Recovering language function and physical ability |
| **11** | F | 17.5 y | 4 months after ACI  Recurrent syncope for 1 month | RA | 92 | Myxoma | None | 1.0 y | None | Lost to follow |
| **12** | M | 17.6 y | Hemoptysis for twice  TR (moderate) | RA | 70 | Myxoma | TV valvuloplasty | 5.0 y | None | NYHA class I |
| **13** | M | 18.0 y | Incidentally found  Asymptomatic | LA | 58 | Myxoma | None | 4.8 y | None | NYHA class I |
| **14** | M | 22 d | Incidentally found  PDA (4 mm)  Asymptomatic | A3 area of MV | 10 | Lipoma | PDA ligation | 1.3 y | None | Normal growth and development |
| **15** | M | 2.0 y | VSD/PH  LVOTO (V_max_ 3.9m/s) | LVOT | 7 | Lipoma | VSD closure | 9.0 y | Reoperation for LVOTO (V_max_ 5.7m/s) after 9 years | In hospital  Eventless recovering |
| **16** | M | 20 d | Found by fetal Echo  Premature birth (33w)  Respiratory failure (intubation)  PDA (5 mm) | RA | 25 | Capillary hemangioma | PDA ligation | 3.0 y | None | Normal growth and development |
| **17** | M | 1 d | Found by fetal Echo  Premature birth (36 w)  PE (large, 13 mm±)  SVC obstruction | RA | 78 | Papillary angioendothelioma (DabsKa tumor) | SVC reconstruction | 7 d | Intracranial hemorrhage | Death |

ACI, acute cerebral infarction; LA, left atria; LV, left ventricle; LVOTO, LV outflow tract obstruction; MV, mitral valve; MR, mitral regurgitation; MS, mitral stenosis; NYHA, New York Heart Association; PDA, patent ductus arteriosus; PH, pulmonary hypertension; PE, pericardial effusion; RA, right atria; RV, right ventricle; RVOTO, RV outflow tract obstruction; SVC, superior vena cava; ST, sinus tachycardia; TV, tricuspid valve; TR, tricuspid regurgitation; VSD, ventricular septal defect.

**^a^** Tumor length is the largest dimension of the mass measured by two-dimensional ultrasound performed within one month prior to operation, and the size descripted in operation records in one patient with intraoperatively found myxoma.

**^b^** V_max_ is the maximum blood flow velocity measured by Doppler ultrasound.

**^c^** Postoperative LV dysfunction is defined as TTE-measured LVEF <50%.

**Supplemental Table 5. Variables associated with postoperative adverse events for patients with primary cardiac tumors (n=39)**

| **Variables** | ***P* value** | | | |
| --- | --- | --- | --- | --- |
|  | **LV dysfunction ^a^** | **Death ^b^** | **Reoperation ^b^** | **Any adverse**  **events ^b, c^** |
| Gender (male vs. female) | 0.182 | 0.898 | 0.806 | 0.944 |
| Age at operation (continuous) | 0.575 | 0.362 | 0.966 | 0.989 |
| Age ≤1 year (yes vs. no) | 0.521 | 0.106 | 0.950 | 0.621 |
| Preoperative symptomatic (yes vs. no) | 0.428 | 0.501 | 0.343 | 0.863 |
| Tumor histotype |  |  |  |  |
| Myxoma | ref. | ref. | ref. | ref. |
| Fibroma | 0.217 | 0.997 | 0.780 | 0.865 |
| Others | 0.902 | 0.826 | 0.756 | 0.308 |
| Tumor length (continuous) | 0.891 | 0.132 | 0.587 | 0.110 |
| Intramyocardial tumor (yes vs. no) | 0.215 | 0.429 | 0.224 | 0.750 |
| Malignant tumor (yes vs. no) | 0.999 | 0.166 | 0.797 | 0.001 |
| CPB time (continuous) | 0.014 | 0.089 | 0.291 | 0.019 |
| Partial resection (yes vs. no) | 0.999 | 0.644 | 0.668 | 0.567 |
| Transmural resection (yes vs. no) | 0.008 | 0.166 | 0.820 | 0.070 |
| Associated procedure (yes vs. no) | 0.762 | 0.897 | 0.721 | 0.698 |

LV, left ventricular; CPB, cardiopulmonary bypass; Ref., references.

LV dysfunction was defined as LV ejection fraction <50% measured by echocardiography. Hemodynamic impairment was defined as mild or greater intracardiac obstruction and/or valvular insufficiency evaluated by echocardiography. Variable with a *P* value <0.20 became candidate for multivariate analysis.

**^a^** *P* value calculated in univariate Logistics regression.

**^b^** *P* value calculated in univariate Cox regression.

**^c^** Any adverse events except for transient postoperative LV dysfunction.

**Supplemental Table 6.** **Univariate logistics regression analysis of variables associated with LV dysfunction after cardiac fibroma resection (n=15)**

| **Variables** | ***P* value** |
| --- | --- |
| Gender (male vs. female) | 0.207 |
| Age at operation (continuous) | 0.742 |
| Age ≤1 year (yes vs. no) | 0.771 |
| Preoperative symptomatic (yes vs. no) | 0.999 |
| Located in Left ventricle (yes vs. no) | 0.771 |
| Tumor length (continuous) | 0.249 |
| Tumor volume index (continuous) | 0.165 |
| Tumor volume index > LVEDVI (yes vs. no) | 0.062 |
| CPB time (continuous) | 0.074 |
| Partial resection (yes vs. no) | 0.999 |
| Transmural resection (yes vs. no) | 0.029 |
| Associated procedure (yes vs. no) | 0.999 |

LVEDVI, left ventricular end-diastolic volume index; CPB, cardiopulmonary bypass.

Variable with a *P* value <0.20 became candidate for multivariate analysis.
